# Bajijiasu Abrogates Osteoclast Differentiation via the Suppression of RANKL Signaling Pathways through NF-κB and NFAT

**DOI:** 10.3390/ijms18010203

**Published:** 2017-01-19

**Authors:** Guoju Hong, Lin Zhou, Xuguang Shi, Wei He, Haibin Wang, Qiushi Wei, Peng Chen, Longkai Qi, Jennifer Tickner, Li Lin, Jiake Xu

**Affiliations:** 1 The National Key Discipline and the Orthopedic Laboratory, Guangzhou University of Chinese Medicine, Guangzhou 510006, China; gdsthgj@gmail.com (G.H.); hewei1123@21cn.com (W.H.); hipknee@163.com (H.W.); weiqshi@126.com (Q.W.); docchen777@gmail.com (P.C.); 2 School of Pathology and Laboratory Medicine, The University of Western Australia, Nedlands WA 6009, Australia; 20463692@student.uwa.edu.au (L.Z.); jennifer.tickner@uwa.edu.au (J.T.); 3 Orthopedic Department, The First Affiliated Hospital of Guangzhou University of Chinese Medicine, Guangzhou 510006, China; 4 College of Chinese Materia Medical, Guangzhou University of Chinese Medicine, Guangzhou 510006, China; sxg6902@126.com (X.S.); booboy@126.com (L.Q.); 5 Artepharm, Co., Ltd., Guangzhou 510000, China

**Keywords:** Bajijiasu, osteoclast, RANKL, NF-κB, NFAT pathway

## Abstract

Pathological osteolysis is commonly associated with osteoporosis, bone tumors, osteonecrosis, and chronic inflammation. It involves excessive resorption of bone matrix by activated osteoclasts. Suppressing receptor activator of NF-κB ligand (RANKL) signaling pathways has been proposed to be a good target for inhibiting osteoclast differentiation and bone resorption. Bajijiasu—a natural compound derived from *Morinda officinalis* F. C. How—has previously been shown to have anti-oxidative stress property; however, its effect and molecular mechanism of action on osteoclastogenesis and bone resorption remains unclear. In the present study, we found that Bajijiasu dose-dependently inhibited RANKL-induced osteoclast formation and bone resorption from 0.1 mM, and reached half maximal inhibitory effects (IC_50_) at 0.4 mM without toxicity. Expression of RANKL-induced osteoclast specific marker genes including cathepsin K (Ctsk), nuclear factor of activated T-cells cytoplasmic 1 (NFATc1), tartrate resistant acid phosphatase (TRAcP), vacuolar-type H^+^-ATPase V0 subunit D2 (V-ATPase d2), and (matrix metalloproteinase-2 (MMP2) was inhibited by Bajijiasu treatment. Luciferase reporter gene studies showed that Bajijiasu could significantly reduce the expression and transcriptional activity of NFAT as well as RANKL-induced NF-κB activation in a dose-dependent manner. Further, Bajijiasu was found to decrease the RANKL-induced phosphorylation of extracellular signal-regulated kinases (ERK), inhibitor of κB-α (IκB-α), NFAT, and V-ATPase d2. Taken together, this study revealed Bajijiasu could attenuate osteoclast formation and bone resorption by mediating RANKL signaling pathways, indicative of a potential effect of Bajijiasu on osteolytic bone diseases.

## 1. Introduction

Osteolytic bone diseases result from the unbalanced activities of osteoblast-induced bone formation and osteoclast-induced bone resorption [1]. Disruption of normal bone metabolic processes can lead to bone diseases, including Paget’s disease of bone, osteonecrosis, erosive arthritis, aseptic bone loosening, and osteoporosis [2,3,4]. Serious complications can develop in osteolytic bone diseases such as skeletal debility, resulting in low impact fractures. Current treatments focus on inhibiting osteoclast formation and function and can limit bone loss, but have unwanted side effects such as osteonecrosis of the jaw, hence the development of novel safe and effective treatments is necessary.

Enhanced osteoclast number and/or function is a key mediator of the pathologic changes observed in osteolytic bone disease. Stimulation and differentiation of osteoclasts is modulated by the receptor activator of nuclear factor-κB (NF-κB) (RANK) ligand (RANKL), which is an essential cytokine in bone metabolic processes [4]. The interaction of RANKL and RANK directly activates a cascade of intracellular molecular events, leading to osteoclast formation and activation. RANKL-activated pathways include NF-κB, mitogen-activated protein kinases (MAPK) including extracellular signal-regulated kinases (ERK), PI3K (phosphatidylinositol-3-kinase) and ionic calcium and calcium/calmodulin-dependent kinase [5]. These sequential molecular events can result in the induction of nuclear factor of activated T-cells cytoplasmic 1 (NFATc1) [6]. Elucidating the molecular mechanisms and potential pharmacological intervention pathways within the bone micro-environment are critical for identifying new therapeutic compounds [7]. We have focused on compound discovery through screening natural herbs to identify novel inhibitors of osteoclast formation and function, and determining their associated pharmacological intervention pathways [8,9].

*Morinda officinalis* F. C. How, also named Bajitian, is one of the best-known herbs in Asian countries, especially in the south of China. It is a natural herb that contains many potential active ingredients including hexasaccharides, oligosaccharides, and anthraquinones [10,11]. Previous studies have demonstrated that anthraquinone can inhibit osteoclastic bone resorption and osteoclast formation, accompanied with reduced expression of calcitonin receptor and carbonic anhydrase (CA) II [12]. Other studies reported the use of alcohol extracts of *Morinda officinalis* root, containing anthraquinones and polysaccharides can increase trabecular bone mineralisation, and increase the expression levels of OPG, tartrate resistant acid phosphatase (TRAcP), and ACTH [13,14]. In addition, *Morinda officinalis*-derived monotropein was found to protect bone loss induced by estrogen deficiency [15], suggesting the therapeutic potential of *Morinda officinalis* in osteolytic bone disease.

**Figure 1:**
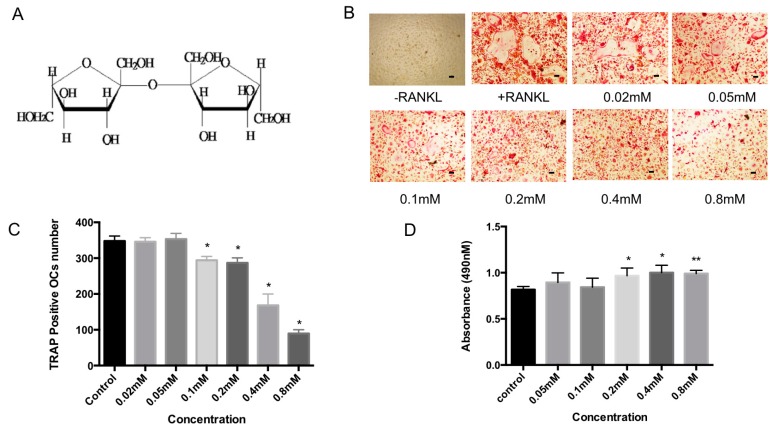
(**A**) Chemical structure of Bajijiasu; (**B**) Representative image of 96 well-plate showing the effects of increasing concentration of Bajijiasu (0.02, 0.05, 0.1, 0.2, 0.4, 0.8 mM) on bone marrow macrophage (BMM)-derived osteoclast-like cell formation, Scale bar = 100 μm; (**C**) Analysis of number of tartrate resistant acid phosphatase (TRAcP)-positive multinucleated (>3 nuclei) cells formed in the presence of increasing concentrations of Bajijiasu; and (**D**) Effect of Bajijiasu on cell survival as assessed by MTS assay (*n* = 3, * *p* < 0.05, ** *p* < 0.01, versus receptor activator of NF-κB ligand (RANKL)-treated control).

Bajijiasu (previously known as Bajisu), also isolated from *Morinda officinalis*, has a dimeric fructose structure (β-d-fructofuranosyl (2-2) β-d-fructofuranosyl (Figure 1A). Previous research indicates that Bajijiasu is absorbed through passive diffusion in the general intestinal segments with the first-order kinetic process. No organ toxicity of the compound had been identified in the kidneys, pituitary gland, or thyroid gland based on histopathological examination [16]. Bajijiasu has been shown to exhibit anti-oxidative stress property [17,18], but its effect and molecular mechanism of action on osteoclastogenesis and bone resorption is unknown.

In search of these new compounds that exhibit pharmacologically inhibitory effects on osteoclasts, we identified Bajijiasu as a compound candidate that can inhibit osteoclastogenesis and bone resorption. Further, we explored the underlying molecular mechanisms by which Bajijiasu modulates RANKL-induced NFAT and NF-κB activation, and ERK phosphorylation.

## 2. Results

### 2.1. Bajijiasu Inhibits RANKL-Induced Osteoclastogenesis

Osteoclastogenesis assays were performed to examine the effect of Bajijiasu on the formation of osteoclasts. Treatment with Bajijiasu resulted in a dose-dependent inhibition of osteoclast formation with an IC_50_ of 0.4 mM (Figure 1B,C). Cell viability was assessed using an MTS (3-(4,5-dimethylthiazol-2-yl)-2,5-diphenyltetrazolium bromide)-based cell proliferation assay (Figure 1D). When bone marrow macrophages (BMM) were cultured with Bajijiasu there were no observed cytotoxic effects up to a dose of 0.8 mM. Thus, the inhibitory effect of Bajijiasu on osteoclast formation was not due to its toxicity.

### 2.2. Bajijiasu Suppresses RANKL-Induced Osteoclast Function

**Figure 2:**
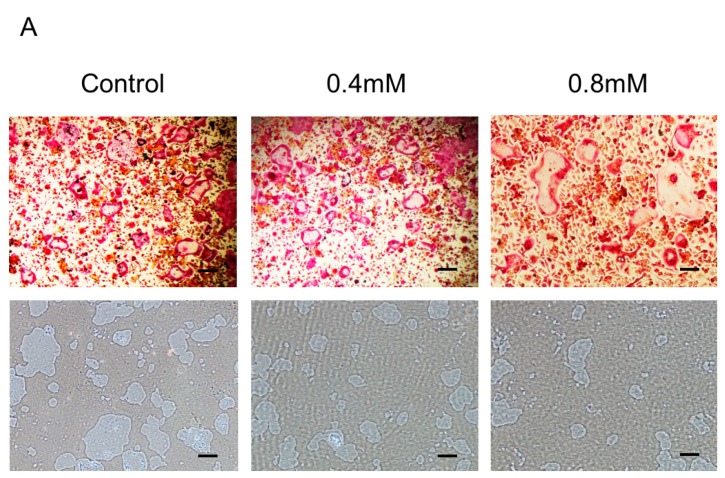

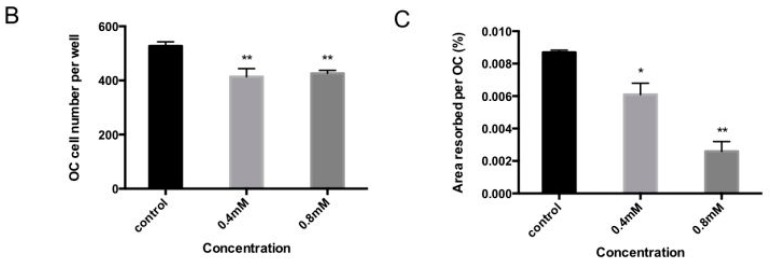
(**A**) Bajijiasu suppresses RANKL-induced hydroxyapatite resorption. Representative images of hydroxyapatite resorption corresponding to TRAcP-stained osteoclasts, Scale bar = 500 μm; (**B**) The number of TRAcP positive multinucleated cells following treatment with Bajijiasu (nuclei ≥ 3); and (**C**) Percentage of the area of hydroxyapatite surface resorbed per osteoclast following treatment with Bajijiasu (*n* = 3, * *p* < 0.05, ** *p* < 0.01, compared with control group).

To address the direct effect of Bajijiasu on mature osteoclast resorptive function, BMM-derived mature osteoclasts were seeded on hydroxyapatite-coated plates and then treated with Bajijiasu for 48 h. Analysis of hydroxyapatite resorption showed that the percentage of area resorbed per osteoclast was significantly attenuated in the presence of Bajijiasu at the concentration of 0.4 and 0.8 mM, compared to the untreated control group (Figure 2). A small, non-dose-dependent, reduction in osteoclast number was observed at both doses tested, indicating that Bajijiasu may also have a small impact on mature osteoclast survival; however, its effects on resorption far exceeded the reduction in mature cell number.

### 2.3. Bajijiasu Suppresses RANKL-Induced Osteoclast-Associated Gene Expression

**Figure 3:**
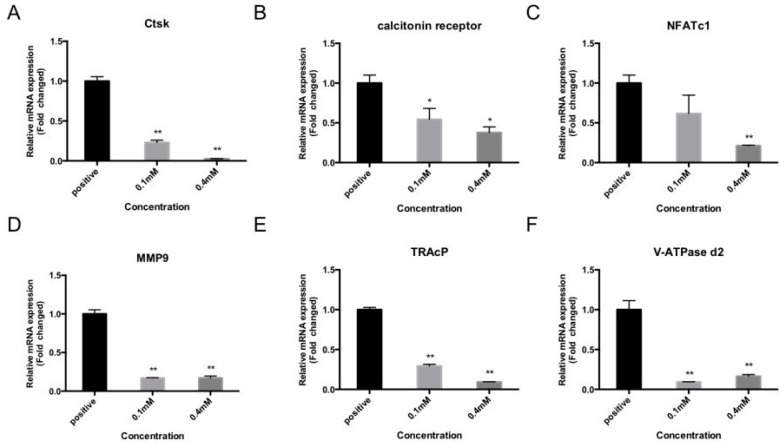
Bajijiasu attenuated RANKL-induced gene expression. RT-pPCR was used to measure the relative levels of RANKL-induced gene expression in BMM cells cultured in the presence of Bajijiasu at 0.1 and 0.4mM. Gene expression was normalized to GAPDH; (**A**) cathepsin K (Ctsk); (**B**) Calcitonin receptor; (**C**) nuclear factor of activated T-cells cytoplasmic 1 (NFATc1); (**D**) MMP2 (matrix metalloproteinase-2); (**E**) tartrate resistant acid phosphatase (TRAcP); and (**F**) vacuolar-type H^+^-ATPase V0 subunit D2 (V-ATPase d2). (*n* = 3, * *p* < 0.05, ** *p* < 0.01 relative to RANKL-stimulated controls).

Activation of NF-κB and NFAT transcriptional activity results in osteoclast specific gene expression. Marker genes, including cathepsin K (Ctsk), NFATc1, TRAcP, MMP9, calcitonin receptor, and vacuolar-type H^+^-ATPase V0 subunit D2 (V-ATPase d2), are necessary for osteoclast formation and bone resorption. To assess the impact of Bajijiasu via inhibition of NF-κB and NFAT transcriptional activity real-time PCR analysis was performed on BMMs that were stimulated with RANKL for five days in the presence of Bajijiasu at 0.1 and 0.4 mM. Our results showed that Bajijiasu strongly, dose-dependently, inhibits mRNA levels of cathepsin K (Ctsk), calcitonin receptor, NFATc1, MMP2, TRAcP, and V-ATPase d2 (Figure 3). This result is consistent with the inhibitory effect of Bajijiasu on osteoclastogenesis and transcriptional activation of NF-κB and NFAT.

### 2.4. Bajijiasu Suppresses RANKL-Induced NF-κB and NFAT Transcriptional Activation

**Figure 4:**
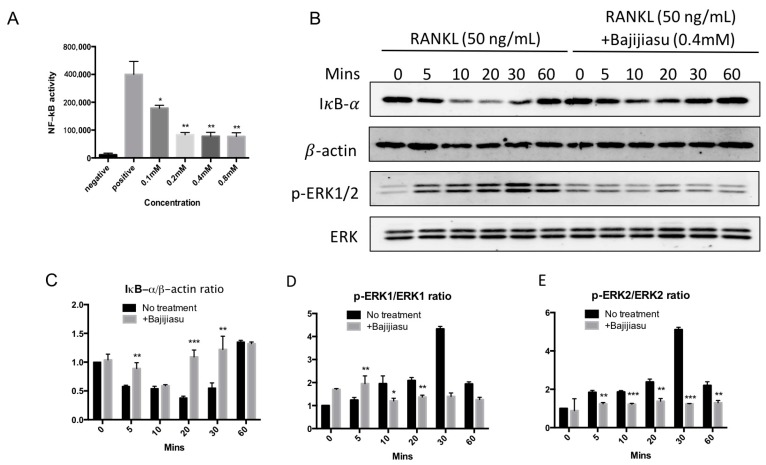
Bajijiasu suppresses RANKL-induced NF-κB activity and proteins of IκB-α and phosphorylation of extracellular signal-regulated kinases (ERK). (**A**) Luciferase activity in RANKL stimulated NF-κB transfected RAW264.7 cells under an luciferase construct. Cells were added with Bajijiasu and then stimulated with glutathione *S*-transferase (GST)-rRANKL (50 ng/mL). (* *p* < 0.05, ** *p* < 0.01 relative to RANKL-stimulated controls); (**B**) Protein lysates from BMMs-induced ostoclasts pre-treated with Bajijiasu (0.4 mM), which were stimulated with GST-rRANKL (50 ng/mL) at given times. Western blot assay was test IκB-α, β-actin and p-ERK, ERK specific antibodies. Relative results were expressed by the ratio of the amount of IκB-α and β-actin (**C**), p-ERK1/ERK1 (**D**) or p-ERK2/ERK2 (**E**) determined by Image J. (* *p* < 0.05, ** *p* < 0.01, *** *p* < 0.001, relative to RANKL-treated, Bajijiasu-untreated controls).

**Figure 5:**
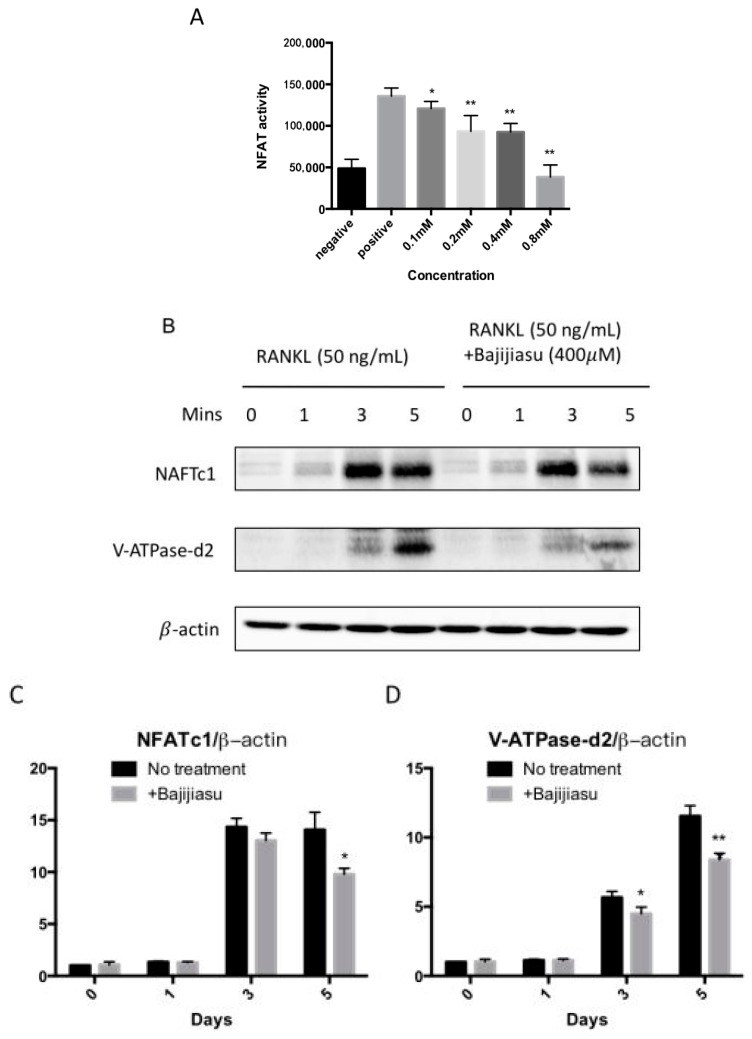
Bajijiasu abrogates RANKL-induced NFATc1 translocate onto nuclei and its protein expression. (**A**) Luciferase activity in RANKL stimulated transfected NFAT RAW264.7 under NFAT luciferase construct. Cells were added with different concentrations of Bajijiasu, and subsequently stimulated with GST-rRANKL (50 ng/mL) (* *p* < 0.05, ** *p* < 0.01 relative to RANKL-stimulated controls); (**B**) Protein lysates from BMMs-induced ostoclasts pre-treated with Bajijiasu (0.4 mM), which were stimulated with GST-rRANKL at given times. Western blot assay was test NFATc1, V-ATPase-d2, and β-actin specific antibodies; and Relative results were expressed by the ratio of the amount of NFATc1/β-actin or VATPase-d2/β-actin determined by Image J (**C**,**D**). (* *p* < 0.05, ** *p* < 0.01, relative to RANKL-treated, Bajijiasu-untreated controls).

NF-κB and NFAT are the key transcriptional mediators regulating osteoclast formation and function. To examine the RANKL-induced NF-κB and NFAT transcriptional activity in the presence of Bajijiasu, luciferase reporter gene assays were performed. The results demonstrated that Bajijiasu strongly inhibited NF-κB transcriptional activity from a dose of 0.1 mM, with an IC_50_ at about 0.2 mM (Figure 4A). Bajijiasu also significantly inhibited RANKL-induced NFAT transcriptional activation from a dose of 0.1 mM, but with reduced efficacy, resulting in an IC_50_ around 0.4 mM (Figure 5A).

### 2.5. Bajijiasu Inhibits RANKL-Induced IκB-α, P-ERK, NFATc1 and V-ATPase d2 Protein Expression

To further clarify the possible molecular mechanism of Bajijiasu on RANKL-induced NF-κB, MAPK and NFAT signaling pathways Western blot assay was utilized. The effects of Bajijiasu on expression and phosphorylation of several key proteins within these pathways, including I-κBα, P-ERK, V-ATPase d2 and NFATc1, was assessed. Degradation of IκB-α is a key downstream step following RANKL engagement with RANK. We found that IκB-α degradation was suppressed by Bajijiasu at the concentration of 0.4 mM, with the most significant effects at 20 and 30 min of RANKL stimulation (Figure 4B–E). Phosphorylation of ERK is downstream of RANKL-induced NF-κB activation, and we found that ERK phosphorylation was strongly inhibited by Bajijiasu treatment. NFATc1 and V-ATPase d2 protein expression are upregulated over the course of osteoclast differentiation. We found that the expression of NFATc1 and V-ATPase d2 was significantly inhibited following continuous Bajijiasu treatment at the concentration of 0.4 mM, particularly in day three and five (Figure 5B–D). These results confirm that Bajijiasu has an inhibitory effect on the expression of key proteins in the RANKL-induced NF-κB, MAPK and NFAT signal pathways.

## 3. Discussion

Excessive bone resorption, due to the imbalance between osteoblast and osteoclast activity, is particularly characteristic in osteolytic bone diseases, including osteoporosis, bone tumors, and osteonecrosis. When considering the molecular mechanisms underlying osteolytic diseases, excessive bone resorption is often caused by increased RANKL levels and subsequent enhancement of its related downstream genes; hence this pathway has been confirmed as a useful target for treatment of osteolytic bone diseases [19,20]. Not restricted to bisphosphonates (BPs) and raloxifene, natural compounds exhibit their potential effects on osteoclastogenesis prohibiting and RANKL signal pathway blocking. Natural compounds have several advantages compared with current anti-osteolytic treatments such as the bisphosphonates and hormone replacement therapies, including low cancer risk, and less gastrointestinal problems [21,22,23]. In this study we have demonstrated that Bajijiasu, a natural compound derived from *Morinda officinalis* F. C. How, is capable of inhibiting osteoclast formation and associated resorption, and inhibiting osteoclast marker gene expression and related protein expression, through blocking RANKL-induced NF-κB and NFAT activities. This indicates that Bajijiasu could be utilized as a therapy to prevent bone destruction in osteolytic bone disease.

*Morinda officinalis* F. C. How has long been utilized in traditional Asian medicine, with minimal side effects, to treat osteolytic bone diseases, like osteoporosis and osteonecrosis. Several isolated compounds from *M. officinalis* F. C. How have been the subject of previous pharmacological investigations into osteoclast suppression such as the anthraquinone compounds [12]. Here, we showed that Bajijiasu a dimeric fructose isolated from the radix of the plant, is one of these novel and promising compounds for the inhibition of osteoclast formation and bone resorption. Recent studies revealed that Bajijiasu has significant role in suppressing oxidative stress [17,18], which confers a protective effect on cell viability and mitochondrial membrane potential in neural cells. Oxidative stress has also been shown to regulate the development of osteoporosis and enhanced osteoclast formation and function [24]. Since RANKL can induce oxidative response [25], it is likely that Bajijiausu have inhibitory effects on RANKL-induced oxidative stress, osteoclastogenesis and bone resoprtion.

Osteoclasts formation is triggered by the differentiation and fusion of macrophage precursors cells [19]. RANKL, belonging to tumor necrosis factor family, is the key regulator of bone loss. RANKL engages with RANK which activates the downstream signaling pathways regulating osteoclast formation [26], including release of NF-κB, phosphorylation of MAPK, and calcium regulated NFATc1 activity [27]. When RANKL and RANK interact, TRAF6 is recruited and actives IKKα and β, which promote IκB-degradation resulting in NF-κB release and activation. NF-κB then translocates into the nucleus and directly activates gene expression [28]. In this study, Bajijiasu inhibited NF-κB transcriptional activity through preventing IκB-α degradation, indicating Bajijiasu acts on IκB-α or its upstream regulators to suppress NF-κB activity. Although the exact Bajijiasu targets are not clear, pivotal downstream pathways, like MAPK and NFATc1 all depend upon the above mechanisms, and are consistently inhibited in our study.

Bajijiasu reduced RANKL-induced phosphorylation of MAPKs, which is regarded as another important pathway modulated by, but not dependent on, NF-κB. Previous studies demonstrated that blockage of ERK1/2 influence osteoclast formation, but notably seldom related with osteoclast performance [29,30]. Thus, inhibiting MAPKs is generally relevant to decreasing osteoclastogenesis [31]. Bajijiasu was found to reduce the phosphorylation of p-ERK1/2. Thus, the suppressive effect of Bajijiasu on RANKL-induced osteoclastogenesis and bone resorption could be mediated, at least in part by the suppression of p-ERK. We assumed that MAPKs pathway was not the primary and original target of Bajijiasu acting on. Bajijiasu might suppress the RANKL up-regulated pERK1/2 under unclear mechanisms, promoting the osteoclast formation, not totally on MAPKs pathway. Hence, further investigation is deserved in the future.

NFATc1 is a master transcription factor modulating osteoclast differentiation [32,33]. Its activation is dependent upon downstream NF-κB activation, and NFATc1 nuclear translocation is also mediated by activated calcium signaling [34]. NFATc1 gene expression is also promoted by NFAT itself, in an automated feedback loop. Several osteoclast-specific genes are regulated by NFATc1 transcriptional activation, such as Ctsk, TRAcP, and V-ATPase d2. NF-κB and NFATc1 improved those genes’ expression for cells differentiation and function requirement [35]. In our study, we found that RANKL-induced NFATc1 transcriptional activity and NFATc1 protein levels were dramatically inhibited by Bajijiasu. This inhibition occurred in a dose-dependent manner; however, the mechanism is still not clear. It is possible that there is a direct intervention in the NFATc1 activation pathway or that Bajijiasu suppression of NF-κB activation directly impacted downstream NFATc1 activity. Regardless of the mechanism Bajijiasu reduced mRNA expression of osteoclast associated proteins, Ctsk, NFATc1, TRAcP, V-ATPase d2, and MMP2. Which are downstream of NFATc1 activity. The change in their expression under Bajijiasu intervention is likely due to suppression of NFATc1 transcriptional activity. These results suggest that Bajijiasu inhibits osteoclast differentiation through both the NF-κB and the NFATc1-mediated pathways. Regarding the role of Bajijiasu on osteoblasts, it is well established that Bajijiasu has some effects on osteoblasts and bone formation [13,14,15]. Thus, Baijijiasu might have a dual effect on pro-osteoblast and anti-osteoclast functions.

In conclusion, our study has identified that Bajijiasu, isolated from the root of *M. officinalis* F. C. How, is able to reduce osteoclast formation and bone resorption through interfering with RANKL-induced NF-κB and NFATc1 activation. Collectively, this study has provided new insight into the potential use of Bajijiasu as an anti-resorptive agent for the treatment of osteolytic bone diseases.

## 4. Materials and Methods

### 4.1. Materials

#### 4.1.1. Reagents and Antibodies

Bajijiasu (Purity > 98%) was extracted and donated by College of Chinese Material Medical, Guangzhou University of Chinese Medicine (Guangzhou, China). The compound was extracted from *Morinda officinalis* root and its purity was analyzed by a high performance liquid chromatography (HPLC) as previously described. For in vitro studies, Bajijiasu was dissolved in fetal bovine serum (FBS) at a stock concentration of 10 mM, and further diluted to working concentrations in tissue culture medium. α-Modified Minimal Essential Medium (α-MEM) and Phosphate buffered saline (PBS) were purchased from Life Technologies (Sydney, Australia). MTS reagent and luciferase analysis reagents were purchased from Promega (Sydney, Australia). Primary antibodies for ERK, phosphorylated ERK, β-actin, IκB-α and NFATc1 were obtained from Santa Cruz Biotechnology (Dallas, CA, USA). All antibodies were used at the concentrations recommended by the supplier at 1:1000. Tartrate resistant acid phosphatase (TRAcP) enzymatic activity was detected using the Leukocyte acid phosphatase staining kit (Sigma, St. Louis, MO, USA). Glutathione *S*-transferase (GST)-rRANKL160–318 (GST-rRANKL) recombinant protein was synthesized and purified by Jiake Xu as previously described [36]. All procedures involving mice were performed according to protocols approved by University of Western Australia Institutional Animal Ethics Committee (RA/3/100/1244) and in accordance with the Australian code for the care and use of animals for scientific purposes 8th edition (2013).

#### 4.1.2. Cell Culture

RAW264.7 cells (mouse macrophage cells) were obtained from America Type Culture Collection (Mannassasa, VA, USA) and maintained in complete α-MEM (α-MEM, 10% heat inactivated FBS, 2 mM l-glutamine and 100 U/mL penicillin/streptomycin). Freshly isolated bone marrow macrophages (BMM) were isolated from C57BL/6 mice by flushing the marrow from the femur and tibia. Primary BMMs were seeded in complete α-MEM supplemented with macrophage-colony stimulating factor (M-CSF), purchased from R&D Systems, (Minnneapolis, MN, USA). All cell cultures were maintained in 5% CO_2_ at 37 °C.

### 4.2. Compound Screening Assay and In Vitro Osteoclastogenesis Assay

BMMs were seeded at a density of 6 × 10^3^ cell/well onto a 96-well plate and stimulated with M-CSF (25 ng/mL) and GST-rRANKL (50 ng/mL), with or without different concentrations of Bajijiasu (0.02, 0.05, 0.1, 0.2, 0.4, 0.8 mM). Bajijiasu and medium were replaced every 2 days. After 5 days cultures were fixed with 4% paraformaldehyde in PBS for 10 min at room temperature and then washed four times with PBS. Detection of TRAcP activity was performed using the Diagnostic Acid Phosphatase kit and the number of TRAcP positive multinucleated cells (more than three nuclei) were counted using a Nikon Ti-U inverted microscope (Nikon Corporation, Tokyo, Japan).

### 4.3. Cytotoxicity Assay

The potential cytotoxicity of Bajijiasu was assessed using an in vitro cell death detection assay. BMMs were plated at a concentration of 6 × 10^3^ cells per well in 96-well plates in 5% CO_2_ at 37 °C. After an overnight incubation to allow adherence, BMMs were treated with complete α-MEM medium with M-CSF (25 ng/mL) and varying concentrations of Bajijiasu (0.05, 0.1, 0.2, 0.4, 0.8 mM). After culturing for 48 h 20 μL MTS ((3-4,5-dimethylthiazol-2-yl)-5-(3-carboxymethoxyphenyl)-2-(4-sulfophenyl)-2*H*-tetrazolium) solution was added to each well and further incubated for 2 h in 5% CO_2_ at 37 °C. The optical density (OD) was measured at 490 nm wavelength using a BMG plate reader (Thermo Labsystem Multiscan Spectrum, ThermoFisher, Waltham, MA, USA).

### 4.4. Hydroxyapatite Resorption Assay

Osteoclast function was assessed using a hydroxyapatite resorption assay. BMMs were seeded at a concentration of 1 × 10^5^ cells/well in 6-well collagen-coated culture plates in complete α-MEM with M-CSF overnight. Osteoclast formation was then stimulated through the addition of 50 ng/mL GST-rRANKL for 4 days. Once mature osteoclasts were observed cells were gently removed from the wells using cell dissociation solution (Sigma, St. Louis, MO, USA). Mature osteoclasts were then plated onto hydroxyapatite-coated plates (Corning, New York, NY, USA) in equal numbers and treated with Bajijiasu at various concentrations (0.4 and 0.8 mM), in the presence of 50 ng/mL GST-rRANKL and M-CSF for 48 h. Half of the wells were dried for hydroxyapatite resorption visualisation using a Nikon microscope (Nikon Corporation, Tokyo, Japan), while the remaining wells were fixed and stained for TRAcP activity for osteoclast counting. The percentage of hydroxyapatite resorption areas were measured using Image J software and normalized to the number of osteoclasts (National Institutes of Health, Bethesda, MD, USA).

### 4.5. Luciferase Reporter Gene Assay for NF-κB and NFAT

In order to measure the effect of Bajijiasu on NF-κB and NFAT transcriptional activation, luciferase reporter gene assays were used. RAW 264.7 cells, stably transfected with an NF-κB luciferase reporter gene (3κB-Luc-SV40) were seeded into 48-well plates at a density of 1.5 × 10^5^ cell/well [37]. Cells were pre-treated with Bajijiasu at 0.1, 0.2, 0.4, or 0.8 mM concentrations for 1 h, and then stimulated with GST-rRANKL (50 ng/mL) for 6 h. Cells were then lysed and luciferase activity was measured using a Promega Luciferase Assay system (Promega, Sydney, Australia). NFAT activation was evaluated using RAW 264.7 cells transfected with an NFATc1 luciferase reporter construct [38]. Cells in 48 well plates were pre-treated with Bajijiasu (0.1, 0.2, 0.4, or 0.8 mM) for 1 h and then stimulated with GST-rRANKL for 24 h prior to cell lysis and measurement of luciferase activity as described above.

### 4.6. Real Time Polymerase Chain Reaction (Real-Time PCR)

For Real-Time PCR, bone marrow macrophages (BMMs) were cultured in a 6-well plate at a density of 1 × 10^5^ cells per well and then cultured in complete α-MEM with M-CSF (25 ng/mL) and GST-rRANKL (50 ng/mL) supplemented with Bajijiasu at several concentrations (0.1 and 0.4 mM) for five days. Total RNA was isolated from the cells using TRIzol reagent (Life Technologies, Sydney, Australia) and single stranded cDNA was prepared from 1 µg of total RNA using MMLV reverse transcriptase with oligo-dT primer (Promega). PCR reactions used specific primers following the mouse sequences:

Cathepsin K (Ctsk) (forward: 5′-GGGAGAAAAACCTGAAG-3′; reverse: 5′-ATTCTGGGGACTCAGAGAGC-3′); TRAcP (Acp5) (forward: 5′-TGTGGCCATCTTTATGCT-3′; reverse: 5′-GTCATTTCTTTGGGGCTT-3′); NFATc1 (forward: 5′-CAACGCCCTGACCACCGATAG-3′; reverse: 5′-GGCTGCCTTCCGTCTCATAGT-3′); V-ATPase d2: (forward: 5′-ATGCTTGAGACTGCAGAG-3′; reverse: 5′-TTATAAAATTGGAATGTAGCT-3′); calcitonin receptor (forward: 5′-TGGTTGAGGTTGTGCCCA-3′; reverse: 5′-CTCGTGGGTTTGCCTCATC-3′); MMP2 (forward: 5′-CGTGTCTGGAGATTCGACTTGA-3′; reverse: 5′-TTGGAAACTCACACGCCAGA-3′); GAPDH (forward: 5′-ACCACAGTCCATGCCATCAC-3′; reverse: 5′-TCCACCACCCTGTTGCTGTA-3′).

Polymerase chain reaction amplification of specific sequences was performed using the following cycle: 94 °C for 5 min, 30 cycles of 94 °C for 40 s, 60 °C for 40 s, 72 °C for 40 s, and finally 72 °C for 5 min. Reaction products were separated using agarose gel electrophoresis and visualized on an Image-quant LAS 4000 (GE Healthcare, Silverwater, Australia).

### 4.7. Western Blot Assay

BMMs were seeded in 12-well plates at a density of 5 × 10^5^ cells per well. Cells were then pretreated with 0.4 mM Bajijiasu for 1 h and then stimulated with GST-rRANKL (50 ng/mL) for the stated times. Cells were then lysed with radioimmunoprecipitation (RIPA) Lysis Buffer (Millipore, Billerica, MA, USA) on ice, and fragments pelleted by centrifugation (14,000× *g* for 5 min). Cleared lysates were transferred into a fresh tube and protein concentrations measured using BCA assay (Promega). Equal amounts of protein samples were then mixed with SDS-polyacrylamide gel electrophoresis (SDS-PAGE) loading buffer and heated at 99 °C for 5 min. Samples were then loaded onto a 10% acrylamide gel and separated using SDS-PAGE. Separated samples were transferred onto a nitrocellulose membrane (GE Healthcare, Silverwater, Australia) and non-specific binding blocked by incubation in 5% skim milk powder diluted in 1× TBS-Tween (TBST) for 1 h at room temperature. Membranes were then incubated with specific primary antibodies (1:1000) at 4 °C, with shaking, overnight. Primary antibody binding was detected using horseradish peroxidase conjugated secondary antibodies (1:5000) coupled with enhanced chemiluminescence (ECL) reagents (Amersham Pharmacia Biotech, Sydney, Australia) and visualized on an Image-quant LAS 4000 (GE Healthcare, Silverwater, Australia).

### 4.8. Statistical Analysis

All original experimental data are reported as mean ± standard deviation (SD). Results are presented as averages or representative values derived from three independent experiments. The Student’s *t*-test was used to assess statistical differences, and a *p*-value < 0.05 was considered to be statistically significant.

## References

[1] Kurihara N. (1990). The origin of osteoclasts. Bull. Kanagawa Dent. Coll..

[2] Kerachian M.A., Seguin C., Harvey E.J. (2009). Glucocorticoids in osteonecrosis of the femoral head: A new understanding of the mechanisms of action. J. Steroid Biochem. Mol. Biol..

[3] Manolagas S.C., Jilka R.L. (1995). Bone marrow, cytokines, and bone remodeling. Emerging insights into the pathophysiology of osteoporosis. N. Engl. J. Med..

[4] Boyle W.J., Simonet W.S., Lacey D.L. (2003). Osteoclast differentiation and activation. Nature.

[5] Baud’huin M., Duplomb L., Ruiz Velasco C., Fortun Y., Heymann D., Padrines M. (2007). Key roles of the OPG-RANK-RANKL system in bone oncology. Expert Rev. Anticancer Ther..

[6] Takayanagi H. (2007). The role of NFAT in osteoclast formation. Ann. N. Y. Acad. Sci..

[7] Whelan A.M., Jurgens T.M., Bowles S.K. (2006). Natural health products in the prevention and treatment of osteoporosis: Systematic review of randomized controlled trials. Ann. Pharmacother..

[8] Tan Z., Cheng J., Liu Q., Zhou L., Kenny J., Wang T., Lin X., Yuan J., Quinn J.M., Tickner J. (2017). Neohesperidin suppresses osteoclast differentiation, bone resorption and ovariectomised-induced osteoporosis in mice. Mol. Cell. Endocrinol..

[9] Song F., Zhou L., Zhao J., Liu Q., Yang M., Tan R., Xu J., Zhang G., Quinn J.M., Tickner J., Huang Y., Xu J. (2016). Eriodictyol Inhibits RANKL-Induced Osteoclast Formation and Function Via Inhibition of NFATc1 Activity. J. Cell. Physiol..

[10] Li Y.F., Yuan L., Xu Y.K., Yang M., Zhao Y.M., Luo Z.P. (2001). Antistress effect of oligosaccharides extracted from *Morinda officinalis* in mice and rats. Acta Pharmacol. Sin..

[11] Li Y.F., Liu Y.Q., Yang M., Wang H.L., Huang W.C., Zhao Y.M., Luo Z.P. (2004). The cytoprotective effect of inulin-type hexasaccharide extracted from Morinda officinalis on PC12 cells against the lesion induced by corticosterone. Life Sci..

[12] Bao L., Qin L., Liu L., Wu Y., Han T., Xue L., Zhang Q. (2011). Anthraquinone compounds from *Morinda officinalis* inhibit osteoclastic bone resorption in vitro. Chem. Biol. Interact..

[13] Li N., Qin L.P., Han T., Wu Y.B., Zhang Q.Y., Zhang H. (2009). Inhibitory effects of morinda officinalis extract on bone loss in ovariectomized rats. Molecules.

[14] Wu Y.B., Zheng C.J., Qin L.P., Sun L.N., Han T., Jiao L., Zhang Q.Y., Wu J.Z. (2009). Antiosteoporotic activity of anthraquinones from *Morinda officinalis* on osteoblasts and osteoclasts. Molecules.

[15] Zhang Z., Zhang Q., Yang H., Liu W., Zhang N., Qin L., Xin H. (2016). Monotropein isolated from the roots of *Morinda officinalis* increases osteoblastic bone formation and prevents bone loss in ovariectomized mice. Fitoterapia.

[16] Wu Z.Q., Chen D.L., Lin F.H., Lin L., Shuai O., Wang J.Y., Qi L.K., Zhang P. (2015). Effect of bajijiasu isolated from *Morinda officinalis* F. C. how on sexual function in male mice and its antioxidant protection of human sperm. J. Ethnopharmacol..

[17] Chen D.L., Zhang P., Lin L., Zhang H.M., Deng S.D., Wu Z.Q., Ou S., Liu S.H., Wang J.Y. (2014). Protective effects of bajijiasu in a rat model of Aβ_25–35_-induced neurotoxicity. J. Ethnopharmacol..

[18] Chen D.L., Ning L., Li L., Deng S.D., Zhang H.M., Liu S.H. (2013). Method to detect the variants of the erythrocyte in a rat model of Aβ_25–35_-induced neurotoxicity based on micro-Raman spectroscopy. J. Biomed. Opt..

[19] Shinohara M., Takayanagi H. (2007). Novel osteoclast signaling mechanisms. Curr. Osteoporos. Rep..

[20] Kong Y.Y., Yoshida H., Sarosi I., Tan H.L., Timms E., Capparelli C., Morony S., Oliveira-dos-Santos A.J., Van G., Itie A. (1999). OPGL is a key regulator of osteoclastogenesis, lymphocyte development and lymph-node organogenesis. Nature.

[21] Newman D.J., Cragg G.M. (2007). Natural products as sources of new drugs over the last 25 years. J. Nat. Prod..

[22] Rachner T.D., Khosla S., Hofbauer L.C. (2011). Osteoporosis: Now and the future. Lancet.

[23] Lippuner K. (2012). The future of osteoporosis treatment—A research update. Swiss Med. Wkly..

[24] Baek K.H., Oh K.W., Lee W.Y., Lee S.S., Kim M.K., Kwon H.S., Rhee E.J., Han J.H., Song K.H., Cha B.Y. (2010). Association of oxidative stress with postmenopausal osteoporosis and the effects of hydrogen peroxide on osteoclast formation in human bone marrow cell cultures. Calcif. Tissue Int..

[25] Yip K.H., Zheng M.H., Steer J.H., Giardina T.M., Han R., Lo S.Z., Bakker A.J., Cassady A.I., Joyce D.A., Xu J. (2005). Thapsigargin modulates osteoclastogenesis through the regulation of RANKL-induced signaling pathways and reactive oxygen species production. J. Bone Miner. Res..

[26] Lacey D.L., Timms E., Tan H.L., Kelley M.J., Dunstan C.R., Burgess T., Elliott R., Colombero A., Elliott G., Scully S. (1998). Osteoprotegerin ligand is a cytokine that regulates osteoclast differentiation and activation. Cell.

[27] Boyce B.F., Xing L., Franzoso G., Siebenlist U. (1999). Required and nonessential functions of nuclear factor-κB in bone cells. Bone.

[28] Nakagomi D., Suzuki K., Nakajima H. (2012). Critical roles of IκB kinase subunits in mast cell degranulation. Int. Arch. Allergy Immunol..

[29] Asagiri M., Takayanagi H. (2007). The molecular understanding of osteoclast differentiation. Bone.

[30] Nakamura H., Hirata A., Tsuji T., Yamamoto T. (2003). Role of osteoclast extracellular signal-regulated kinase (ERK) in cell survival and maintenance of cell polarity. J. Bone Miner. Res..

[31] Ikeda F., Nishimura R., Matsubara T., Tanaka S., Inoue J., Reddy S.V., Hata K., Yamashita K., Hiraga T., Watanabe T. (2004). Critical roles of c-Jun signaling in regulation of NFAT family and RANKL-regulated osteoclast differentiation. J. Clin. Investig..

[32] Asagiri M., Sato K., Usami T., Ochi S., Nishina H., Yoshida H., Morita I., Wagner E.F., Mak T.W., Serfling E. (2005). Autoamplification of NFATc1 expression determines its essential role in bone homeostasis. J. Exp. Med..

[33] Aliprantis A.O., Ueki Y., Sulyanto R., Park A., Sigrist K.S., Sharma S.M., Ostrowski M.C., Olsen B.R., Glimcher L.H. (2008). NFATc1 in mice represses osteoprotegerin during osteoclastogenesis and dissociates systemic osteopenia from inflammation in cherubism. J. Clin. Investig..

[34] Takayanagi H., Kim S., Koga T., Nishina H., Isshiki M., Yoshida H., Saiura A., Isobe M., Yokochi T., Inoue J. (2002). Induction and activation of the transcription factor NFATc1 (NFAT2) integrate RANKL signaling in terminal differentiation of osteoclasts. Dev. Cell.

[35] Feng H., Cheng T., Steer J.H., Joyce D.A., Pavlos N.J., Leong C., Kular J., Liu J., Feng X., Zheng M.H., Xu J. (2009). Myocyte enhancer factor 2 and microphthalmia-associated transcription factor cooperate with NFATc1 to transactivate the V-ATPase d2 promoter during RANKL-induced osteoclastogenesis. J. Biol. Chem..

[36] Xu J., Tan J.W., Huang L., Gao X.H., Laird R., Liu D., Wysocki S., Zheng M.H. (2000). Cloning, sequencing, and functional characterization of the rat homologue of receptor activator of NF-κB ligand. J. Bone Miner. Res..

[37] Wang C., Steer J.H., Joyce D.A., Yip K.H., Zheng M.H., Xu J. (2003). 12-*O*-tetradecanoylphorbol-13-acetate (TPA) inhibits osteoclastogenesis by suppressing RANKL-induced NF-κB activation. J. Bone Miner. Res..

[38] Van der Kraan A.G., Chai R.C., Singh P.P., Lang B.J., Xu J., Gillespie M.T., Price J.T., Quinn J.M. (2013). HSP90 inhibitors enhance differentiation and MITF (microphthalmia transcription factor) activity in osteoclast progenitors. Biochem. J..

